# Supplementary material for: Paternal methotrexate exposure affects sperm small RNA content and causes craniofacial defects in the offspring
Source: Nat Commun. 2023 Mar 23;14:1617. doi: 10.1038/s41467-023-37427-7 (PMC10036556; doi:10.1038/s41467-023-37427-7)
Supplement: Supplementary file 1 — Supplementary Information [file 41467_2023_37427_MOESM1_ESM.pdf]

Supplementary Figures

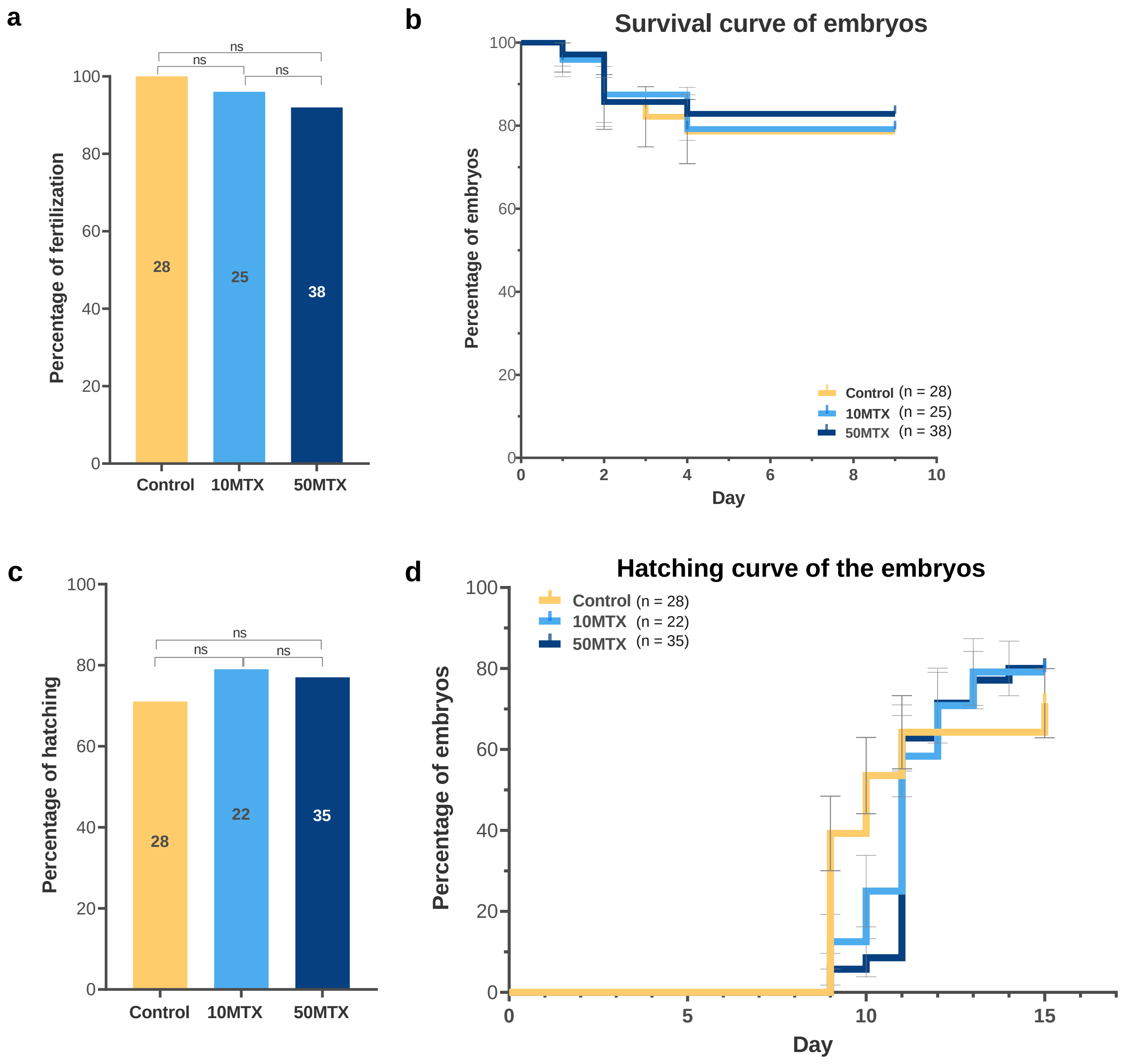

Figure S1: (a) Histogram showing the percentage of fertilization utilizing the sperm from control and MTX treated males. **(b)** Kaplan Meier plot displaying the survival of fertilized eggs until the beginning of hatch. Histogram showing the percentage of hatching embryos **(c)** and the day of hatching **(d)** on control and MTX group. Values are means ± SEM. Numbers in the graph represent the analyzed embryos. Statistics for A and C were generated by contingency table followed by Chi-square test. Statistics for B and D were generated by using the Long-rank (Matel-Cox) test, Gehan-Breslow-Wilcoxon test. ns: P > 0.05.

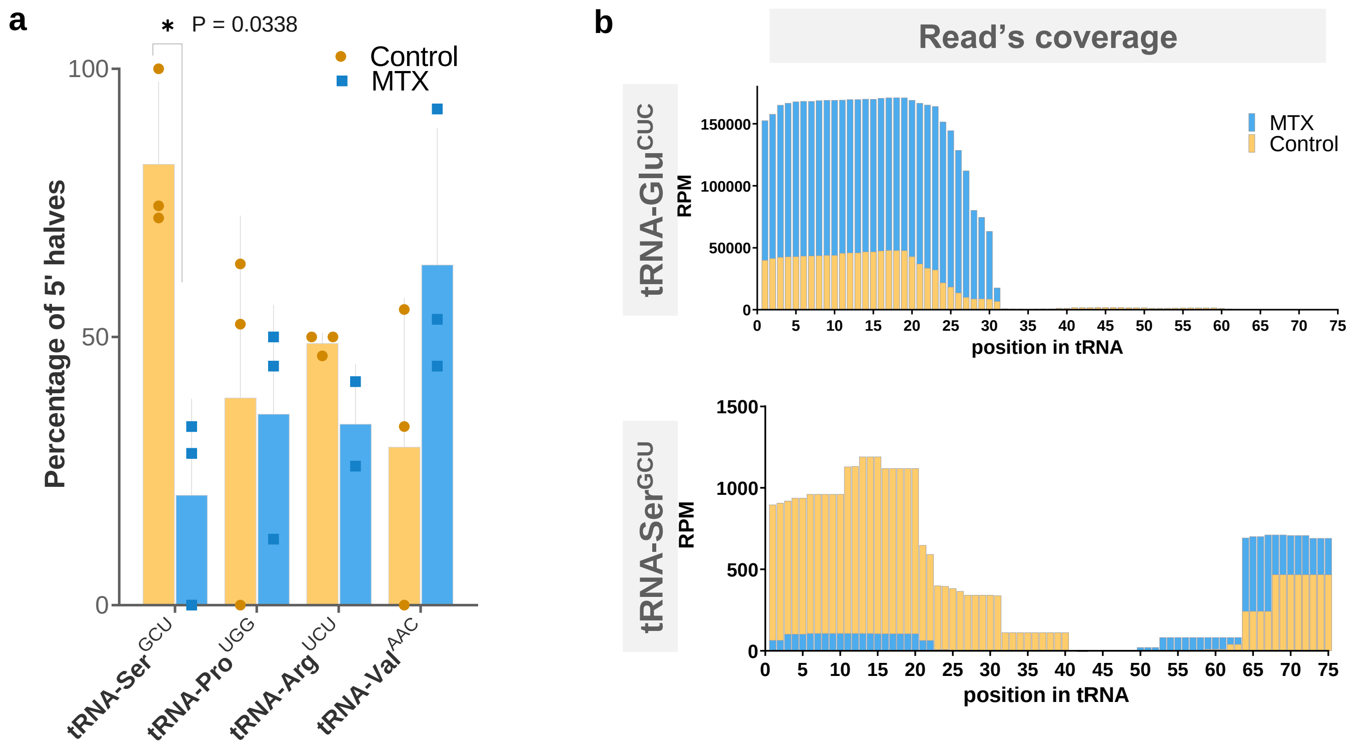

Figure S2: (a) Histogram displaying percentage of 5' halves relative to their corresponding 3' halves from tRNAs not affected (tRNA-Pro^{UGG}, -Arg^{UCU}, -Val^{AAC}) or having a reduction (tRNA-Ser^{GCU}) on MTX treatment. Data represent three biologically independent replicates (n = 3) composed by RNA isolated from 9 males' sperm. Asterisk indicated significant differences analyzed by multiple unpaired t-student' test followed by a correction for multiple comparison (Holm-Sidak method, with alpha = 0.05). Values are means ± SD. **(b)** Histogram showing the read coverage for tRNA-Glu^{CUC} and -Ser^{GCU}. See also **Supplementary Data 5**.

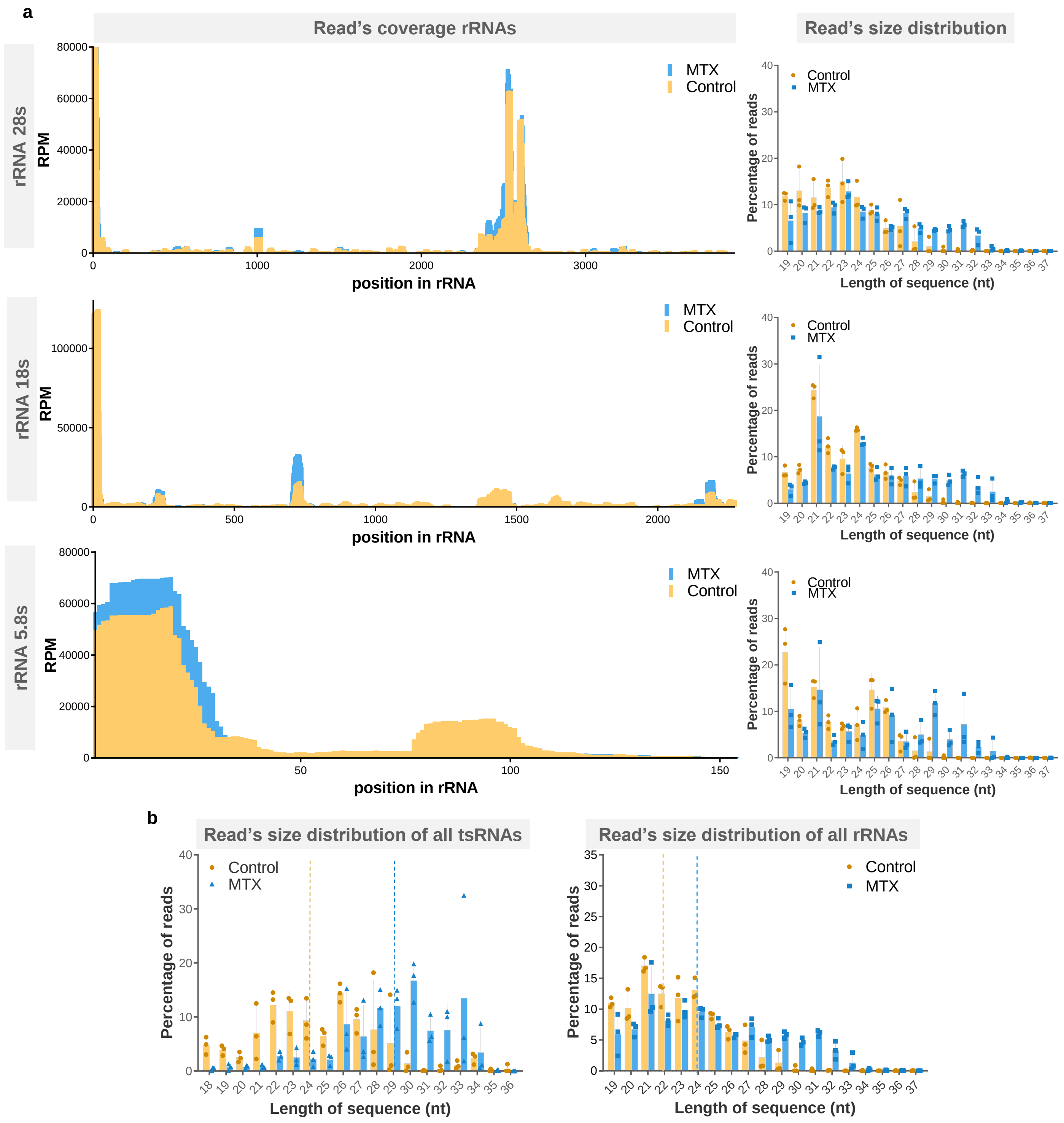

Figure S3: (a) Histogram showing the read coverage and size distribution for the most abundant rRNAs (28s, 18s and 5.8s) between control and MTX. **(b)** Histograms showing the size distribution for all mapped tsRNA and rRNA on control and MTX treated males. Dotted lines represent the median length. Values on read's size distribution are means ± SD. See also **Supplementary Data 5**.

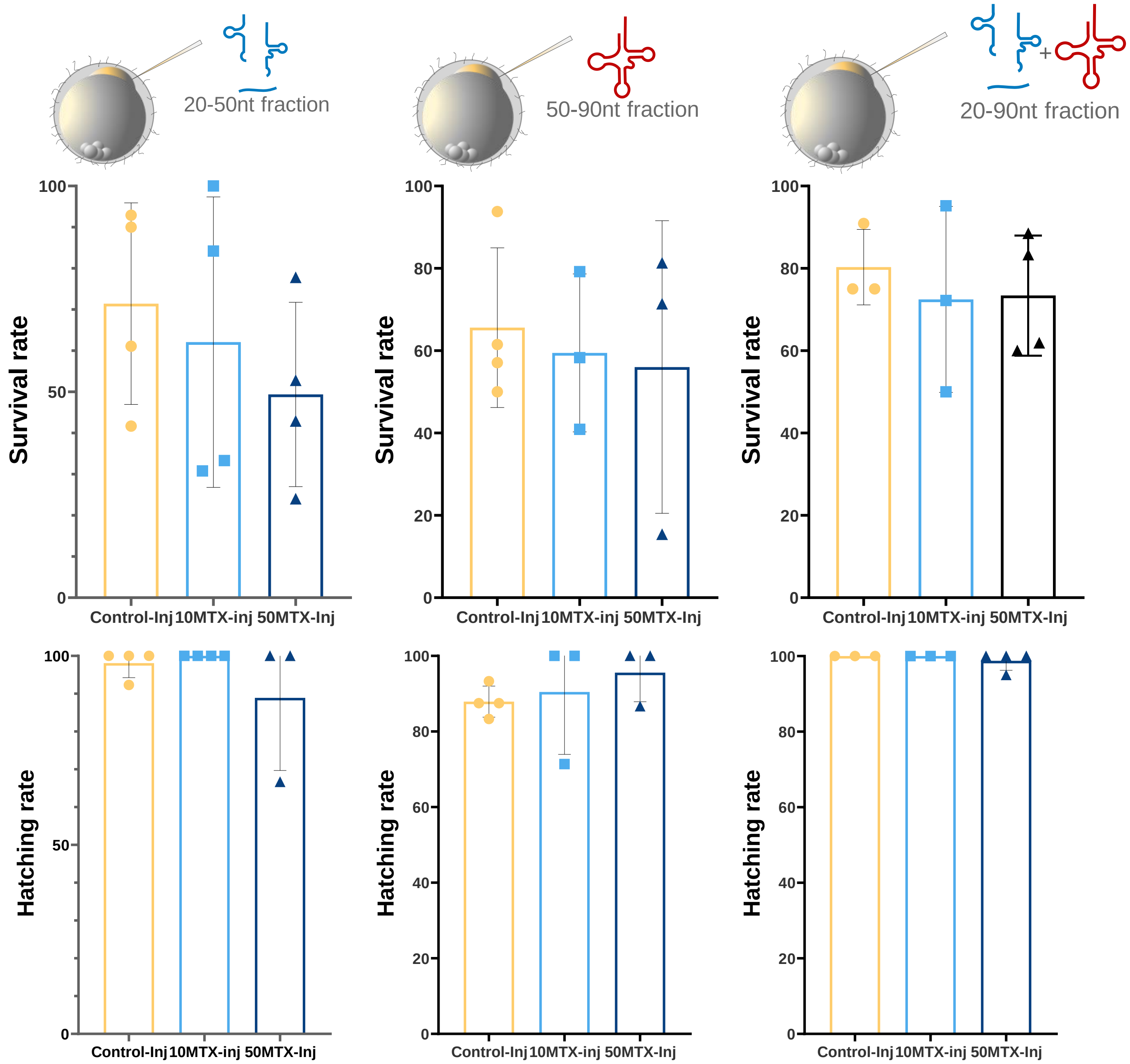

Figure S4: Histogram showing the percentage of survival and hatching of different batches of fertilized wild-type eggs injected with sperm RNA fractions obtained from control, 10MTX and 50MTX treated males.). Each dot represents a biological experimental replicate composed by a pool of embryos. Number of embryos utilized: 20-50nt RNA Control-Inj (n= 14, 24, 18, and 20); 10MTX-Inj (n= 26, 18, 20, and 38); and 50MTX-Inj (n= 9, 14, 25, and 36). 50-90nt RNA Control-Inj (n= 16, 13, 12, 14, and 12); 10MTX-inj (n= 24, 22, 12, and 22); and 50MTX-Inj (n= 16, 21, and 39). 20-90nt RNA Control-Inj (n= 11, 20, and 28); 10MTX-Inj (n= 21, 18, and 16); and 50MTX-Inj (n= 24, 26, 15, and 21).

Hatching rate; for 20-50nt RNA Injections: Control-Inj: n=13, 10, 11, 18; 10MTX-Inj: n= 8, 6, 20, 32: and 50MTX-Inj: n=7, 6, 6 viable embryos at the hatching start point. For 50-90 nt RNA Inj: Control-Inj: n= 15, 8, 6, 8, 11; 10MTX-Inj: n= 19, 9, 7, 18; 50MTX-Inj: n= 13, 15, 6 viable embryos at the hatching start point. For 20-90nt RNA injection: Control-Inj: n= 10, 15, 21; 10MTX-Inj: n= 20, 13, 8; 50MTX-Inj: n= 20, 23, 9, 13 viable embryos at the hatching start point. Statistics by one-way ANOVA followed by Turkey's multiple comparisons test. Non-significant differences were observed in all cases ($P > 0.05$). Values are means ± SE.

Supplementary Tables

Gene symbol	Accession Number	Primer sequence (5´- 3´)
Trmt6	ENSORLG00000009862.2	**Fw:** CGGGAACAGACAACAGGAATA<br>**Rv:** GACCCTTCAGACCTTGATCTTT
Dnmt2 (Trdmt1)	ENSORLG00000019403.2	**Fw:** AGGCCACCTCGCTTTATTC<br>**Rv:** CTTGTGGGAGAGGCCATAATC
Nsun2	ENSORLG00000014332.3	**Fw:**AGATGCTTCACGCTGACATG<br>**Rv:** TTGTTGTCCACGTCATTGGC
RPL7	ENSORLG00000007967.2	**Fw:** CGCCAGATCTTCAACGGTGTAT<br>**Rv:** AGGCTCAGCAATCCTCAGCAT
ef1α	ENSORLG00000007614	**Fw:** GGAGGCCAGCGACAAGATGAGC.<br>**Rv:** ACACGGCCGACAGGGACAGTTC

Supplementary table 1: Primer sequences utilized for RT-qPCR

Sample	Species	3' Adapter used	3' Adapter barcode	3' barcoded adapter	5' Adapter	small-RNAseq RT primer	Solexa_PCR_fwd	Solexa_IDX_rev
**Control-1**	*O. latipes*	SRBC1	CAGTG	5′-/5rApp/NNNNNNCAGTGAGATCGGAAGAGCACACGTCT/3ddC/-3'	5′-ACACUCUUUCCCUACACGACGCUCUUCCGAUCUNNNN-3’	5′-AGACGTGTGCTCTTCCGATCT -3’	5′-AATGATACGGCGACCACCGAGATCTACACTCTTTCCCTACACGACGCTCTTCCGATCT -3’	5′-CAAGCAGAAGACGGCATACGAGATCAGTGGTGACTGGAGTTCAGACGTGTGCTCTTCCGATCT – 3’
**Control-2**	*O. latipes*	SRBC2	AGCAA	5′-/5rApp/NNNNNNAGCAAAGATCGGAAGAGCACACGTCT/3ddC/-3'	5′-ACACUCUUUCCCUACACGACGCUCUUCCGAUCUNNNN-3’	5′-AGACGTGTGCTCTTCCGATCT -3’	5′-AATGATACGGCGACCACCGAGATCTACACTCTTTCCCTACACGACGCTCTTCCGATCT -3’	5′-CAAGCAGAAGACGGCATACGAGATAGCAAGTGACTGGAGTTCAGACGTGTGCTCTTCCGATCT – 3’
**Control-3**	*O. latipes*	SRBC3	GGTAT	5′-/5rApp/NNNNNNGGTATAGATCGGAAGAGCACACGTCT/3ddC/-3'	5′-ACACUCUUUCCCUACACGACGCUCUUCCGAUCUNNNN-3’	5′-AGACGTGTGCTCTTCCGATCT -3’	5′-AATGATACGGCGACCACCGAGATCTACACTCTTTCCCTACACGACGCTCTTCCGATCT -3’	5′-CAAGCAGAAGACGGCATACGAGATGGTATGTGACTGGAGTTCAGACGTGTGCTCTTCCGATCT – 3’
**10MTX-1**	*O. latipes*	SRBC4	TACCA	5′-/5rApp/NNNNNNTACCAAGATCGGAAGAGCACACGTCT/3ddC/-3'	5′-ACACUCUUUCCCUACACGACGCUCUUCCGAUCUNNNN-3’	5′-AGACGTGTGCTCTTCCGATCT -3’	5′-AATGATACGGCGACCACCGAGATCTACACTCTTTCCCTACACGACGCTCTTCCGATCT -3’	5′-CAAGCAGAAGACGGCATACGAGATTACCAGTGACTGGAGTTCAGACGTGTGCTCTTCCGATCT – 3’
**10MTX-2**	*O. latipes*	SRBC5	GTCAG	5′-/5rApp/NNNNNNGTCAGAGATCGGAAGAGCACACGTCT/3ddC/-3'	5′-ACACUCUUUCCCUACACGACGCUCUUCCGAUCUNNNN-3’	5′-AGACGTGTGCTCTTCCGATCT -3’	5′-AATGATACGGCGACCACCGAGATCTACACTCTTTCCCTACACGACGCTCTTCCGATCT -3’	5′-CAAGCAGAAGACGGCATACGAGATGTCAGGTGACTGGAGTTCAGACGTGTGCTCTTCCGATCT – 3’
**10MTX-3**	*O. latipes*	SRBC6	TGACT	5′-/5rApp/NNNNNNTGACTAGATCGGAAGAGCACACGTCT/3ddC/-3'	5′-ACACUCUUUCCCUACACGACGCUCUUCCGAUCUNNNN-3’	5′-AGACGTGTGCTCTTCCGATCT -3’	5′-AATGATACGGCGACCACCGAGATCTACACTCTTTCCCTACACGACGCTCTTCCGATCT -3’	5′-CAAGCAGAAGACGGCATACGAGATTGACTGTGACTGGAGTTCAGACGTGTGCTCTTCCGATCT – 3’

Supplementary Table 2: Adapter oligos utilized for library preparation.
